# A Moment's Reflection

**Published:** 1853-04

**Authors:** 


***A Moment's Reflection.***—This certainly seems to be the "time and tide" for our profession. The old and the young are meeting in friendly fellowship. Men are beginning to feel that in union lies strength, and that kind of strength which is required to elevate professional standing. General intelligence in professional topics is lighting up the path of progress, and hundreds are marching on the high road to fame, honor and success, with a deep and earnest determination to win the laurels that are properly showered upon industrious usefulness.

How the heart kindles when we hear of a valuable scientific or practical achievement of a professional brother, and now we are almost constantly hearing of such. There are many well educated scientific dentists belonging to the profession, scattered abroad all over the country, who are beginning to unite their efforts for the accomplishment of a great common good, and forming a common center of improvement. The beautiful chain of unity is passing through and around our land—uniting our brethren into one body. Many are now so qualified, in intelligence and practical knowledge—in position and acquirements, exhibiting that liberal spirit, which cannot rest without liquidating the debt they owe to the calling of their choice, leading them (as it has done) to an honorable position, exalting and improving the practice of their art, by concentrating its scattered knowledge, and manfully urging the necessity of union for one great object—professional elevation, and by encouraging and establishing upon the firmest foundation that code of preparatory education, without which we must, as a body, ever be considered pretending presumers; and what can be said of the man who is satisfied to labor only for the advancement of his own individual and pecuniary interests, at the sacrifice of the general respectability and usefulness of his calling, allowing his noble art, capable of such immense service to mankind, to fall into obscurity and deterioration.

